# Leptin Resistance in Vagal Afferent Neurons Inhibits Cholecystokinin Signaling and Satiation in Diet Induced Obese Rats

**DOI:** 10.1371/journal.pone.0032967

**Published:** 2012-03-07

**Authors:** Guillaume de Lartigue, Claire Barbier de la Serre, Elvis Espero, Jennifer Lee, Helen E. Raybould

**Affiliations:** Department of Anatomy, Physiology and Cell Biology, School of Veterinary Medicine, University of California Davis, Davis, California, United States of America; Sapienza University of Rome, Italy

## Abstract

**Background and Aims:**

The gastrointestinal hormone cholecystokinin (CCK) plays an important role in regulating meal size and duration by activating CCK1 receptors on vagal afferent neurons (VAN). Leptin enhances CCK signaling in VAN via an early growth response 1 (EGR1) dependent pathway thereby increasing their sensitivity to CCK. In response to a chronic ingestion of a high fat diet, VAN develop leptin resistance and the satiating effects of CCK are reduced. We tested the hypothesis that leptin resistance in VAN is responsible for reducing CCK signaling and satiation.

**Results:**

Lean Zucker rats sensitive to leptin signaling, significantly reduced their food intake following administration of CCK8S (0.22 nmol/kg, i.p.), while obese Zucker rats, insensitive to leptin, did not. CCK signaling in VAN of obese Zucker rats was reduced, preventing CCK-induced up-regulation of Y2 receptor and down-regulation of melanin concentrating hormone 1 receptor (MCH1R) and cannabinoid receptor (CB1). In VAN from diet-induced obese (DIO) Sprague Dawley rats, previously shown to become leptin resistant, we demonstrated that the reduction in EGR1 expression resulted in decreased sensitivity of VAN to CCK and reduced CCK-induced inhibition of food intake. The lowered sensitivity of VAN to CCK in DIO rats resulted in a decrease in Y2 expression and increased CB1 and MCH1R expression. These effects coincided with the onset of hyperphagia in DIO rats.

**Conclusions:**

Leptin signaling in VAN is required for appropriate CCK signaling and satiation. In response to high fat feeding, the onset of leptin resistance reduces the sensitivity of VAN to CCK thus reducing the satiating effects of CCK.

## Introduction

Under normal physiological conditions, the presence of fat in the small intestine stimulates the release of gut hormones, including cholecystokinin (CCK) and peptide YY (PYY) [1], [2], thereby slowing gastric emptying [3] and suppressing food intake [1]. Paradoxically, chronic consumption of a high fat diet (HFD) results in hyperphagia and obesity [4], [5]. There is evidence that obesity in both humans [6] and rats [7] results in reduced sensitivity to the satiating effect of lipids compared to their lean counterparts. This does not appear to occur as a result of reduced release of CCK [2], but rather to a reduction in sensitivity to the satiating effects of CCK [8]–[10]. The mechanisms leading to the decrease in response to CCK remain unknown, although it seems to occur independently of CCK1 receptor (CCK1R) expression [11].

CCK, released from the small intestine in response to fatty acids and proteins, activates CCK1R located on vagal afferent nerve terminals in the gut to inhibit gastric emptying and food intake, and stimulating pancreatic enzyme secretion and gall bladder contraction. In addition to stimulating the discharge of vagal afferent neurons (VAN), CCK also changes the neurochemical phenotype of VAN. The change in neurochemical phenotype is characterized by altered expression levels of G-protein coupled receptors and neuropeptide transmitters. Following feeding or administration of exogenous CCK, the peptide YY type 2 (Y2) receptor [12] and the anorexigenic neuropeptide transmitter cocaine and amphetamine regulated transcript (CART) [13] are upregulated; synthesis of the orexigenic neuropeptide transmitter melanin-concentrating hormone (MCH) [13], [14] as well as the MCH1 receptor (MCH1R) [14] and CB1 receptor (CB1) [15] are inhibited. Conversely, fasting, or feeding in the presence of a CCK1R antagonist, decreases expression of CART and Y2, and increases MCH, MCH1R and CB1 expression in VAN. This suggests that CCK acts as the “gatekeeper” in the control of the neurochemical phenotype in VAN and can influence food intake and GI function both rapidly, by stimulating action potentials, or induce prolonged effects, by regulating the expression of proteins known to play a role in the control of feeding behavior.

The long form of the leptin receptor has been found to be co-expressed with CCK1R in a subset of VAN [16]–[18]. Leptin alone rapidly increases VAN discharge [19], [20] and increases cytosolic calcium in culture [21]. However, leptin also plays a crucial role in modulating the sensitivity of these neurons to CCK. Leptin markedly enhances vagal afferent discharge [20], cytosolic calcium [21], [22], and translocation of the early gene EGR-1 to the nucleus [23] in response to low doses of CCK. Leptin regulates EGR-1 levels while CCK activates EGR-1 in VAN. Inhibition of EGR-1 abolishes leptin-induced sensitization of VAN to CCK and reduces CCK-induced expression of CART in VAN [23]. Vagally-mediated changes in function, such as inhibition of food intake and gastric emptying in response to CCK, are also enhanced by leptin [23], [24].

We have recently demonstrated that chronic ingestion of a high fat diet leads to the development of leptin resistance in VAN of diet-induced obese rats, compared to low fat fed control rats or rats resistant to the obesigenic effects of a high fat diet [4]. In the present study, we hypothesize that leptin resistance in VAN of DIO rats is responsible for the reduced sensitivity of these neurons to CCK, resulting in an altered neurochemical phenotype in VAN and hyperphagia. Firstly, Zucker rats which have a genetic deletion of the leptin receptor were used to determine the effect of altered leptin function on VAN. Secondly, Sprague-Dawley rats with a predisposition to diet-induced obesity (DIO), that develop leptin resistance in VAN when fed a HF diet [4], were used to determine changes in CCK-induced inhibition of food intake and signaling in VAN in response to long term ingestion of a high fat diet.

## Results

### Reduced sensitivity of VAN to CCK in obese Zucker rats

**Figure 1:**
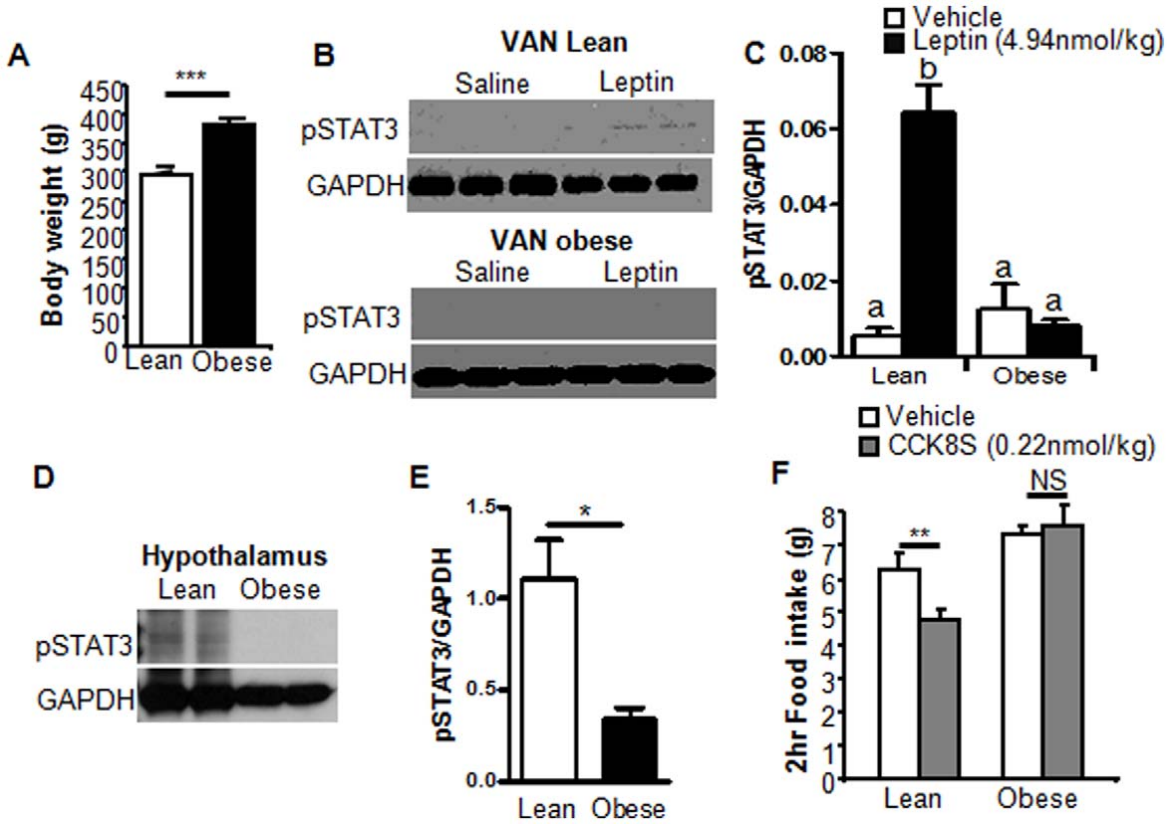
Altered CCK induced satiety in obese Zucker rats. A) Obese Zucker rats weigh significantly more than lean Zucker rats (N = 12; t-test). B) Immunoblot of pSTAT3 in VAN of lean (top) and obese (bottom) Zucker rats treated with vehicle or leptin. C) Densitometry analysis of *B* showing that STAT3 is phosphorylated in response to leptin (i.p) in VAN of lean Zucker rats but not in obese Zucker rats. N = 4 D) Immunoblot of pSTAT3 in hypothalamus of lean and obese Zucker rats treated with leptin. E) Densitometry analysis of *D* showing that STAT3 is phosphorylated in response to leptin in the arcuate nucleus of the hypothalamus of lean Zucker rats, but not obese Zucker rats. (N = 4).F) CCK8S (i.p., 0.22 nmol/kg) significantly inhibited food intake in lean Zucker rats but not obese Zucker rats. N = 6. Data expressed as mean ± SEM. Significant differences were represented as ^a,b,c^ between groups in one-way ANOVA. Significant differences were represented as * for p<0.05; ** for p<0.01; and *** for p<0.001 in Student's t-test.

Initial studies using Zucker rats demonstrated that leptin signaling is required for CCK-induced activation of VAN and its subsequent anorexigenic effects. Zucker rats are a genetic model of leptin receptor deficiency [25], [26]. As previously reported, homozygous Zucker rats (LepR^fa^/LepR^fa^), weighed significantly more than lean Zucker rats when fed on chow (p<0.01; Fig. 1*A*). Exogenous leptin (4.94 nmol/kg; i.p.) phosphorylated STAT3 in the nodose ganglia and arcuate nucleus of lean Zucker rats; however as expected, leptin had no effect on STAT3 phosphorylation in either arcuate or vagal afferent neurons from obese Zucker rats (Fig. 1*B–E*).

To determine whether CCK-induced inhibition of food intake requires leptin signaling, food intake in fasted lean and obese Zucker rats was measured following administration of CCK8S (i.p.; 0.22 nmol/kg). In lean Zucker rats, CCK induced a 25% reduction in food intake compared to saline; CCK had no effect on food intake in obese Zucker rats (Fig. *1F*).

**Figure 2:**
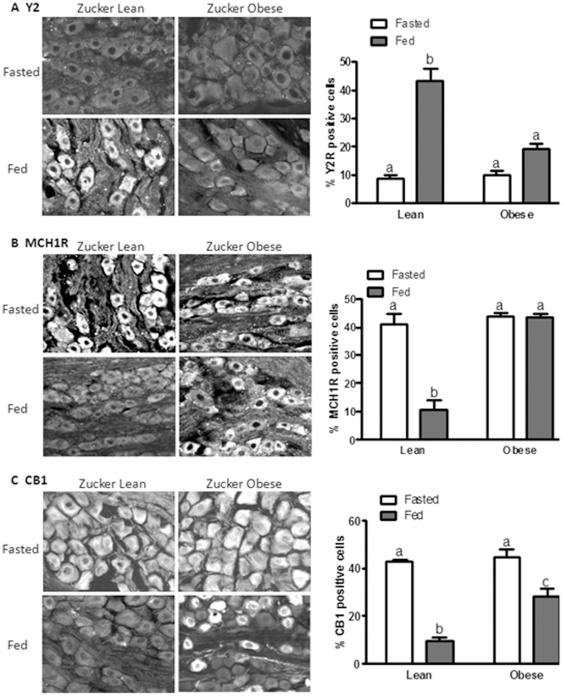
Altered CCK induced signaling in obese Zucker rats. Photomicrographs of sections of nodose ganglia to show immunoreactivity for Y2 (A), MCH1R (B), and CB1 (C) from lean and obese Zucker rats fasted 24 hr or refed for 1 hr. A) Y2 expression is elevated by feeding in lean Zucker rats, but not in obese Zucker rats. N = 4. B) MCH1R is decreased by feeding in lean Zucker rats but not in obese Zucker rats. N = 4. C) CB1 is decreased by feeding in lean and obese Zucker rats, but CB1 expression remains significantly higher in fed obese Zucker rats compared to lean Zucker rats. Significant differences were represented as ^a,b,c^ between groups.

To determine whether leptin signaling is required in the regulation of the neurochemical phenotype of VAN, immunoreactivity of Y2, MCH1R and CB1 was examined in the nodose ganglia of lean and obese Zucker rats in the presence or absence of food. In 24 hour fasted lean Zucker rats, the predominant phenotype of VAN was low levels of Y2 expression and high levels of MCH1R and CB1 (Fig. 2); feeding (which releases CCK) increased Y2 expression and decreased MCH1R and CB1 expression (Fig. 2). In fasted obese Zucker rats, expression levels of Y2, CB1 and MCH1R were similar to that observed in fasted lean Zucker rats; however, feeding failed to up-regulate Y2 expression (Fig. 2*A*, P>0.05), or down-regulate MCH1R expression in VAN (Fig. 2*B*; p>0.05). CB1 expression was down-regulated in VAN of fasted obese Zucker rats (Fig. 2*C*; p<0.05), but was still significantly elevated compared to fasted lean Zucker rats (Fig. 2*C*; p<0.01).

### Effect of a HF diet on body weight and food intake

Sprague Dawley rats carry a polygenic susceptibility to high fat diet-induced obesity; individuals become either diet-induced obese (DIO) or remain lean (diet-induced obese resistant DR) when ingesting high fat diet [27]. We have recently demonstrated that chronic (8 week) ingestion of a high fat diet leads to the development of leptin resistance in VAN of DIO, but not in DR or low fat (LF) fed control rats, and that this occurs in the absence of measurable leptin resistance in the arcuate nucleus of the hypothalamus [4]. Hence this rat model was used to determine whether leptin signaling in VAN is required for CCK-signaling and its subsequent anorexigenic effects.

**Figure 3:**
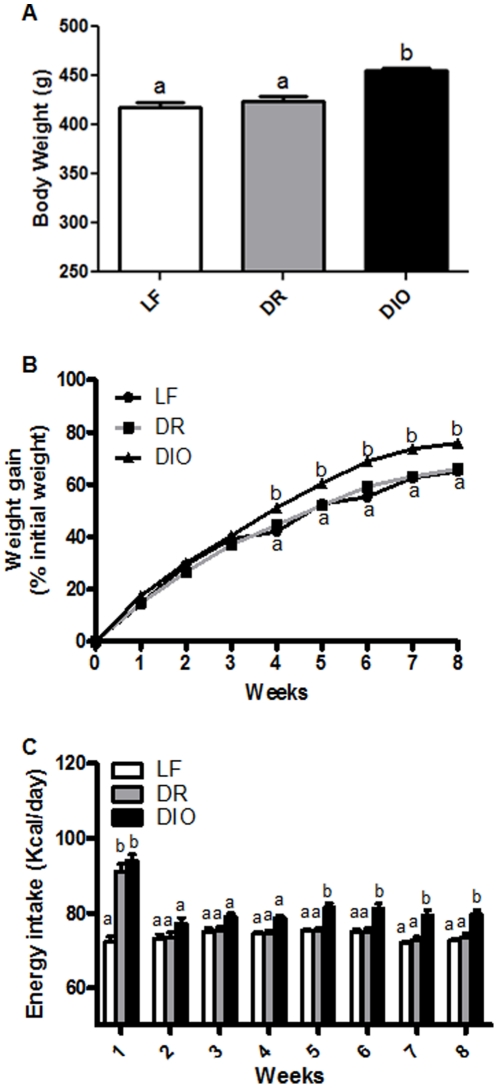
Effect of a high-fat (HF) diet on body weight and food intake *A*) Body weight of rats ingesting either a LF diet or HF diet (45% kcal) for 8 weeks. There was a significant increase in body weight in diet-induced obesity (DIO) rats compared with diet-induced obesity-resistant (DR) and LF animals. *B*) The increase in body weight of LF, DR, DIO rats expressed as a percent of weight gain from initial weight. DIO rats became significantly heavier than LF fed animals after 4 weeks of HF diet, and after 4 weeks of a HF diet compared to the DR rats. *C*) Average daily food intake (kcal) per week over the 8 weeks on the diets. In the first week, all rats on the HF diet had a significantly higher energy intake than LF fed rats but this initial hyperphagia only lasted for one week after which there was no significant difference in caloric intake between the groups. At *week 5*, the DIO group had a significantly higher energy intake than LF rats and DR rats. N = 12 per group; data expressed as mean ± SEM. Significant differences were represented as ^a,b,c^ between groups.

A total of 24 Sprague Dawley rats were fed a HF diet for 8 weeks. Rats with the highest body weight at 8 weeks were assigned to the DIO group (n = 12), the other rats were designated DR (n = 12). After 8 weeks, DIO rats had significantly higher body weight compared to either LF fed controls rats or DR rats (p<0.01, Fig. 3*A*); there was no significant difference between DR and LF controls (*p*>0.05). DIO rats had a significantly higher body weight gain after 4 weeks on a HF diet compared to both the DR and LF controls (p<0.01; p<0.001, respectively; Fig. 3*B*).

In the first week on a HF diet, both DIO and DR groups had a higher caloric intake than LF fed controls (p<0.001). Following acclimation to the HF diet in the first week, DR rats had equal caloric intake to the LF fed controls. DIO rats had a consistently higher caloric intake than either LF and DR rats but this only reached significance after 5 weeks (p<0.001, p<0.01, respectively, Fig. 3*C*).

### Leptin resistance in VAN decreases CCK-induced inhibition of food intake

**Figure 4:**
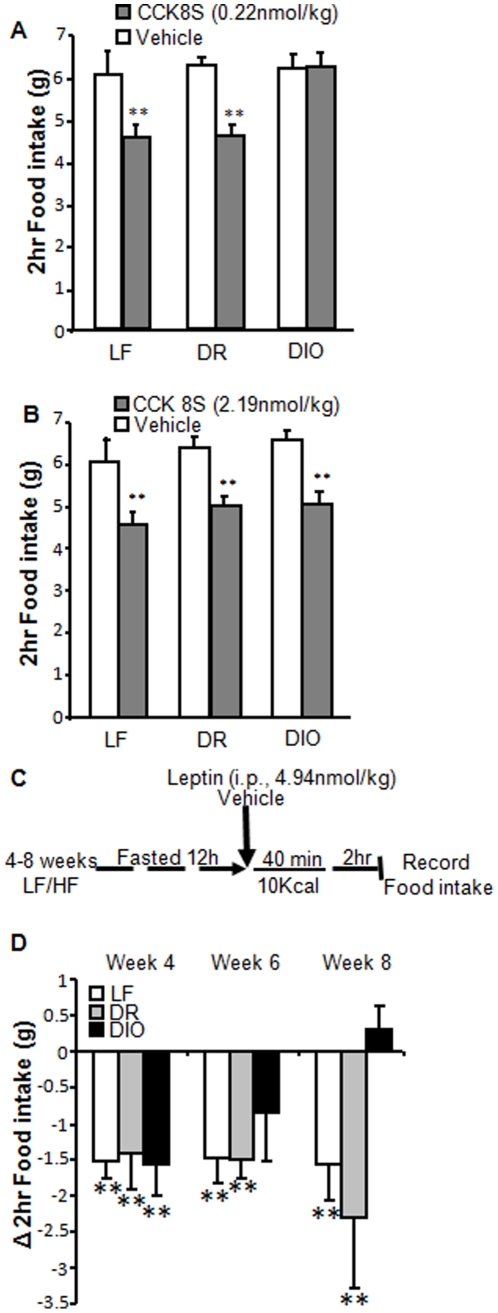
Reduced satiation to exogenous and endogenous CCK in DIO rats. A) CCK feeding study (described in methods) in Sprague Dawley rats after 8 weeks on respective diets. CCK8S (0.22 nmol/kg; i.p.) significantly inhibited food intake compared to vehicle in LF and DR, but not DIO rats. N = 6. B) CCK8S (2.19 nmol/kg; i.p.) significantly inhibited food intake in all rats. N = 6. C) Protocol for leptin feeding study in which endogenous CCK was upregulated D) Endogenous CCK reduced food intake in LF fed and DR rats at 4, 6 and 8 weeks. Endogenous CCK reduced food intake in DIO rats at 4 weeks but not at 6 and 8 weeks. N = 6; data expressed as mean ± SEM. Significant differences were represented as ** for p<0.01.

Administration of CCK8S (i.p., 0.22 nmol/kg) after 12 hr fasting reduced food intake by 27% in LF and DR rats compared to saline (Fig. 4*A*; p<0.01) but had no effect in DIO rats (Fig. 4*A*; p>0.05). However, a higher dose of CCK8S (i.p., 2.19 nmol/kg) significantly reduced food intake in DIO as well as LF and DR rats compared to saline (Fig. 4*B*; p<0.01).

To determine whether leptin resistance in VAN of DIO rats would inhibit the satiating effects of endogenous CCK, we used a previously described feeding protocol in which a small preload meal was used to release endogenous CCK [23]. In this protocol we found that exogenous leptin had no effect on food intake in fasted rats. After a preload meal (which increases endogenous CCK), we observed that exogenous leptin inhibited food intake, and these effects were abolished by a CCK1R antagonist, lorglumide [23]. Therefore, we concluded that leptin potentiates the satiating effects of endogenous CCK. Here we show using this same protocol (Fig. 4*C*) that after 4 weeks on the respective diets, leptin significantly potentiated CCK-induced inhibition of food intake in LF, DR, and DIO rats (LF, p<0.01; DR, p<0.01; DIO, p<0.01; Fig. 4*D*). After 6 weeks on a HF diet, DIO rats receiving leptin (i.p.; 4.94 nmol/kg) no longer significantly inhibited food intake even in the presence of endogenous release of CCK by a preload meal (LF: p<0.01; DR: p<0.01; DIO: p>0.05; Fig. 4*D*). After 8 weeks leptin had no effect on food intake in DIO rats (LF: p<0.01; DR: p<0.01; DIO: p>0.05; Fig. 4*D*). In contrast, LF fed and DR rats after 6 and 8 weeks on LF or HF diets, respectively, had reduced feeding in response to exogenous leptin (i.p..; 4.94 nmol/kg) compared to vehicle following the release of endogenous CCK induced by the preload meal (p<0.01; Fig. 4*D*).

### Leptin resistance in VAN decreases CCK-induced alteration in neurochemical phenotype

**Figure 5:**
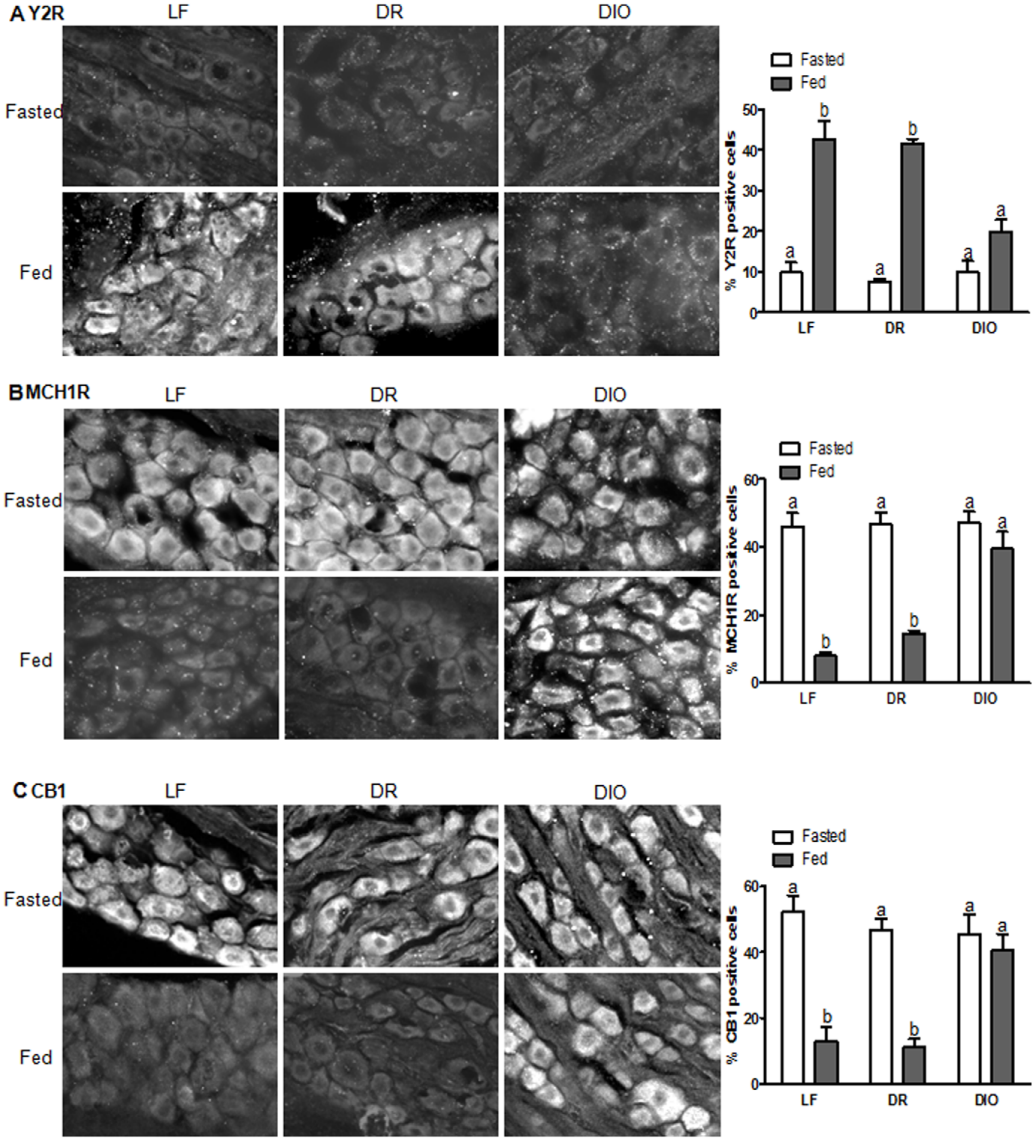
Altered neurochemical phenotype in DIO rats. Photomicrographs of sections of nodose ganglia to show immunoreactivity for Y2 (A), MCH1R (B), and CB1 (C) from fed and fasted Sprague Dawley rats after 8 weeks on respective diets. A) Y2 is barely detectable in nodose neurons of LF, DR, and DIO rats fasted 24 hours. Refeeding for 1 hour increased Y2 expression in LF and DR but not DIO rats. Quantification of positive Y2 cells as a percent of total cells. B) MCH1R and C) CB1 expression is elevated in fasted rats and is decreased by refeeding for 1 hour in LF and DR but not DIO rats. Quantification of positive MCH1R and CB1 cells as a percent of total cells. Representative images from experiments with four to six rats in each group are shown. Significant differences were represented as ^a,b,c^ between groups.

Under fasting conditions, VAN of LF and DR rats expressed low Y2 abundance while in the fed state Y2 abundance is increased as previously described (Fig. 5*A*) [12]. In contrast, Y2 expression was constitutively low in VAN from DIO rats in the fed and fasted states (Fig. 5*A*). Similarly, in LF and DR rats, MCH1R and CB1 expression was high in VAN from fasted rats and decreased upon feeding (Fig. 5*B–C*); however, in DIO rats these proteins were constitutively expressed even in the fed state (Fig. 5*B–C*).

### Leptin resistance prevent CCK signaling in VAN

**Figure 6:**
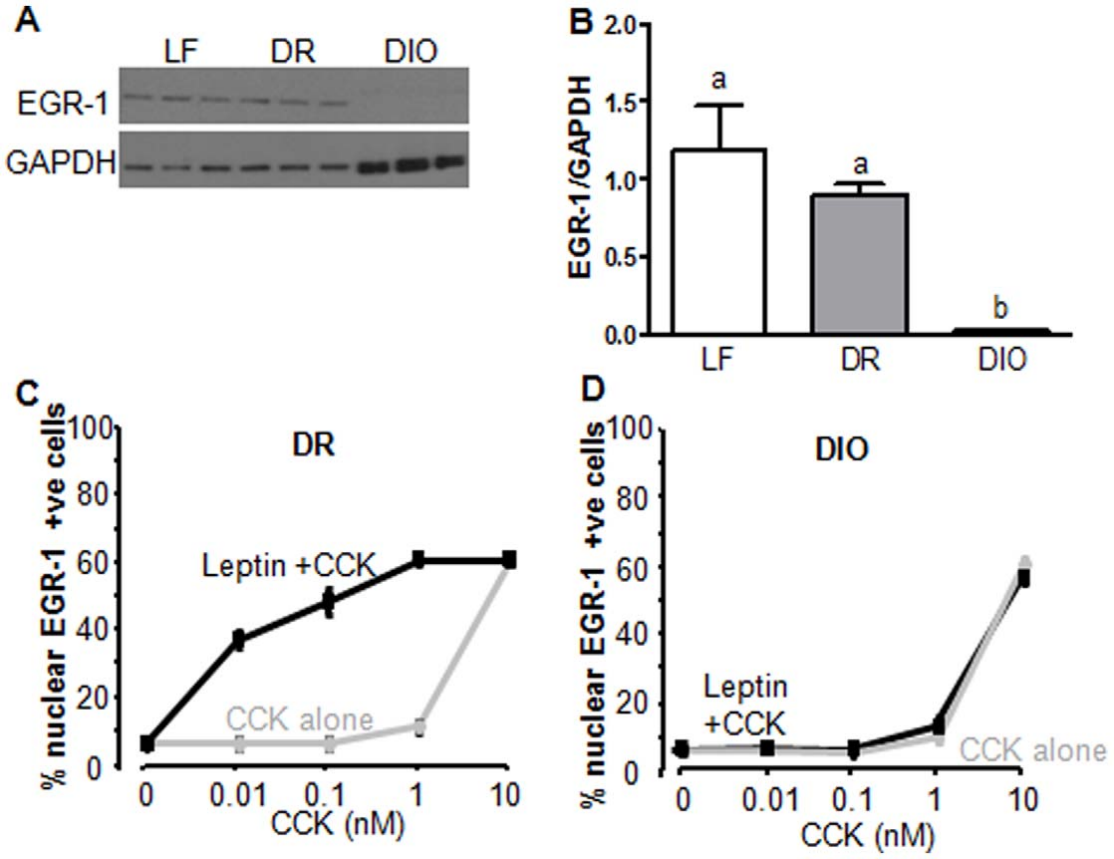
EGR-1 expression in VAN of DIO rats. A) Protein expression of EGR-1 in VANs of LF, DR and DIO rats treated with leptin. B) Densitometry analysis of *A* showing that EGR-1 levels in VAN are significantly reduced in DIO rats compared to LF and DR rats. N = 3. C) and D) Quantification of the number of EGR-1 immunopositive neurons in cultured VAN from DR rats (C) and DIO rats (D) fed a high fat diet for 8 weeks. C) Leptin reduced the concentration of CCK required to induce EGR-1 translocation in cultured VAN. D) Leptin had no effect on CCK induced EGR-1 translocation in cultured VAN. N = 6. Data expressed as mean ± SEM. Significant differences were represented as ^a,b,c^ between groups.

Leptin-induced phosphorylation of the transcription factor STAT3 is required for EGR-1 expression and EGR-1 is involved in mediating gene expression [23]. We have previously shown that leptin resistance (as defined by an absence of phosphorylation of STAT3 in response to leptin) is present in VAN of DIO rats after 8 weeks of chronic high fat feeding [4]. We hypothesized, therefore, that the absence of pSTAT3 in leptin-resistant VAN of DIO rats would result in low EGR-1 abundance. To test this, we measured EGR-1 levels by western blot in the nodose ganglia of leptin-treated rats following 8 weeks on the respective diets. EGR-1 expression was high in VAN of both LF and DR rats in response to leptin (i.p.; 4.94 nmol/kg), but was absent in VAN from DIO rats (Fig. 6*A–B*).

Leptin and CCK interact at the level of EGR1 in VAN. To study these interactions at the cellular level, we cultured VAN of DR and DIO rats and determined the effect of leptin-resistance on CCK-induced EGR-1 translocation to the nucleus. In cultured VAN from DR rats (Fig. 6*C*), CCK8S alone induced maximal EGR-1 activation at 10 nM but had little effect at lower doses. Leptin alone (10 ng/ml) had no effect on EGR-1 nuclear translocation but significantly increased the number of nuclear EGR-1-positive neurons in the presence of low doses of CCK (0.01–1 mM) (Fig. 6*C*). The dose-dependent response to CCK alone in VAN of DIO rats (Fig. 6*D*) was identical to that of VAN from DR (Fig. 6*C*) or LF rats [23]. However, the ability of leptin to act synergistically with CCK in VAN of DIO rats is completely abolished (Fig. 6*D*).

## Discussion

We have previously shown that VAN from diet-induced obese rats are leptin-resistant; this leptin resistance occurs only in rats that express the obese phenotype and not in rats that remain lean when ingesting a HF diet. Here, we wanted to determine the consequences of impaired leptin signaling in VAN by studying EGR-1 signaling in VAN, changes in VAN neurochemical phenotype in response to feeding, and functional responses on control of food intake. The data show that in Zucker rats, the lack of leptin signaling is associated with a complete lack of phenotypic change of VAN normally induced by feeding and this is associated with an absence of CCK-induced inhibition of food intake in these rats. Moreover, in DIO rats, the lack of leptin signaling in VAN, results in a lack of phenotypic change in VAN in response to feeding, a decrease in CCK-induced nuclear EGR-1 and a lack of endogenous CCK-induced inhibition of food intake. Taken together, the data suggest that leptin resistance in VAN may account for the well known decrease in lipid- and CCK-induced satiation and hyperphagia following chronic HF feeding. Inaccurate monitoring of nutrients in the intestinal lumen may lead to a lack of satiety and contribute to diet-induced hyperphagia and obesity.

Leptin acts synergistically with CCK and can modulate CCK signaling in VAN [23], [24], [28] and leptin has also been found to potentiate the inhibitory action of CCK on food intake [23], [24]. Zucker rats are completely insensitive to leptin as a result of a single amino acid substitution of a glutamine for a proline in the leptin receptor gene *(Ob-R)* [*25*], [*29*]. Zucker rats homozygous for the *fa* gene are morbidly obese and characterized by fat cell hypertrophy and hyperplasia [30], increased adipose tissue, lipoprotein lipase activity [31], hyperinsulinemia, hypertriglyceridemia, and hyperphagia [32] compared to their lean heterozygous counterparts. Unsurprisingly, we found that exogenous administration of leptin in these rats failed to phosphorylate STAT3 in both the arcuate nucleus and the nodose ganglia, while lean Zucker rats responded normally to leptin. We demonstrate that loss of leptin signaling in Zucker rats inhibits CCK induced signaling in VAN and at a dose that inhibits food intake in lean Zucker rats, CCK failed to inhibit food intake in obese Zucker rats.

Under normal physiological conditions leptin and CCK interact at the level of EGR1 in VAN. Leptin upregulates expression of EGR1 in these neurons, while CCK activates EGR1. Inhibition of EGR1 abolishes leptin potentiation of CCK-induced protein synthesis in VAN [23]. Here we demonstrate that altered leptin signaling, as a result of VAN resistance to leptin following prolonged ingestion of a HF diet, prevents high EGR1 expression in VAN. To study the interactions between leptin and CCK at the cellular level, we used cultured VAN from DR and DIO rats. Interestingly the dose-dependent translocation of EGR1 to the nucleus in response to CCK alone in VAN of DIO rats was identical to that of VAN from DR rats, demonstrating that the sensitivity of VAN from DIO rats to CCK alone is not changed (Fig. 6*D*). However, the ability of leptin to act synergistically with CCK in the DIO rats is completely abolished. Therefore the response to CCK is unaltered, but rather the potentiating effect of leptin on CCK is lost in VAN of DIO rats, suggesting that leptin activation of VAN is required for optimal CCK signaling.

Similarly, the satiating effects of CCK in DIO rats are not completely abolished, instead higher concentrations of CCK are required in these rats to inhibit food intake. Administration of a low dose of CCK (0.22 nmol/kg) after a 12 hr fast inhibited food intake in LF and DR rats but had no effect in DIO rats. However, a 10-fold higher dose of CCK produced a significant reduction in food intake in both DIO and DR rats. We propose that loss of the potentiating effects of leptin in DIO rats is responsible for the reduced sensitivity VAN to CCK signaling and subsequently that higher concentrations of CCK are required for satiation.

Feeding in the presence of a CCK1 receptor antagonist completely abolishes feeding induced Y2 expression and inhibition of MCH1R and CB1, and injection of CCK in fasted animals increases Y2 and inhibits MCH1R and CB1 [12]–[15], [33], [34] _ENREF_13. Hence, under normal physiological conditions, CCK is required for the regulation of the neurochemical phenotype in VAN [13]. Here we found that lean Zucker rats had high Y2 and low MCH1R and CB1 abundance in VAN; however, in obese Zucker rats, Y2 expression was constitutively low in VAN and feeding failed to inhibit MCH1R and CB1 expression in VAN. Therefore in the absence of leptin signaling, VAN are unable to alter their neurochemical phenotype in response to endogenous CCK after a meal.

Similarly, VAN of DIO rats did not respond to feeding, exhibiting elevated Y2 abundance, as well as reduced MCH1R and CB1 expression compared to LF or DR rats. DIO rats develop leptin resistance in VAN, prior to measurable leptin resistance in the ARC, therefore we conclude that hypothalamic leptin resistance is not required for CCK induced signaling and satiation. We hypothesize instead that reduced VAN sensitivity to CCK, as a result of leptin resistance in these neurons, is responsible for the altered expression of these receptors and would have important functional consequences on vagally mediated regulation of food intake.

Changes in receptor expression on VAN of DIO animals suggest that the sensitivity of VAN to the gastrointestinal hormones that bind these receptors will be altered. Reduced Y2 expression in VAN of obese animals would prevent signaling of the anorexigenic hormone PYY_3–36_ via VAN in response to a meal. PYY_3–36_ is an anorectic peptide released from L cells in response to a meal which inhibits pancreatic enzyme secretion [35], influences gastric emptying [36] and inhibits food intake [37]. Subdiaphragmatic vagotomy attenuates the effects of PYY_3–36_ on food intake in rats [38], [39] suggesting that Y2 receptor downregulation in VAN of obese animals would prevent the satiating effects of PYY_3–36_. Furthermore constitutively high MCH1R and CB1 expression in VAN of obese animals suggests that anandamide released from the gut and MCH from VAN would prolong orexigenic signaling. Thus altered receptor expression could increase the orexigenic tone of the vagus, potentially resulting in hyperphagia.

DIO rats develop hyperphagia between weeks 4 and 5 following chronic HF feeding. In the first 4 weeks, DIO and DR rats consume the same amount of food, but by week 5 the DIO rats consume 10 kcal per day more than the DR rats (Fig. 3*C*). Interestingly we provide evidence that functional leptin resistance develops between weeks 4 and 6 of a HF diet, which coincides with the onset of hyperphagia in these rats (Fig. 4*D*).

There is evidence in the literature to suggest that altered CCK sensitivity could result in hyperphagia. Both knockout mice and OLEFT (Otsuka Long Evans Tokushima Fatty) rats that lack the CCK1R eat larger meals than their respective controls. Crucially OLETF (Otsuka Long Evans Tokushima Fatty) rats, which lack the CCK-1 receptor gene, have increased average meal size, with reduced meal frequency that is insufficient to compensate for the increase in meal size, resulting in hyperphagia [40]. Interestingly, pair feeding OLETF rats with intact controls (LETO rats) prevented the development of obesity [41]. This suggests that reduced sensitivity to CCK results in hyperphagia and could directly lead to the development of obesity.

While mice lacking the CCK_1_R eat larger and longer meals both in response to chow or a high fat diet compared to wild type mice [42], they have statistically insignificant increases in total daily food intake and maintain normal body weight. The reason for the difference in response between knockout mice and OLEFT rats to the absence of CCK_1_R is not clear. However, it should be noted that the knockout mice are on a 129S genetic background, which is known to confer resistance to obesity [43]. Mice with this genetic background have been reported to have low feeding efficiency (small weight gain per calorie consumed), and high basal energy expenditure [44]. Another important difference is that OLEFT and LETO rats express the NPY gene in the dorsal motor nucleus of the hypothalamus (DMH) while wild-type and CCK1 receptor^−/−^ mice do not. Crucially, DMH NPY gene expression is dysregulated in OLEFT rats so that in response to a high-fat diet, NPY expression is significantly reduced in LETO rats but not in OLETF rats [45].

In conclusion, we demonstrate that the onset of leptin resistance in VAN of DIO rats results in the reduced sensitivity of VAN to CCK. This leads to altered expression of receptors in VAN known to play a role in the regulation of food intake. We hypothesize that these changes result in increased orexigenic signaling and reduced anorexic sensing of hormones from the gut resulting in the development of hyperphagia.

## Materials and Methods

### Ethics Statement

All experiments with animals reported complied with relevant national and international guidelines. All experimental procedures involving rats complied with an animal studies protocol (#15202) approved by the UC Davis Institutional Animal Care and Use Committee (IACUC) and PHS animal welfare assurance to UC Davis (#A3433-01).

### Rats

Male Sprague Dawley rats (initial weight 180 g; Harlan San Diego, n = 36) were housed at 22°C under a 12 h light (6am–6pm), 12 h dark (6pm–6am) cycle with *ad libitum* access to food and water. Rats were kept on chow (13% kcal/g fat; Purina 5001) or high fat (HF) diet (45% kcal/g fat; Research Diets D12451) for 8 weeks. Body weight and food intake were measured twice a week. Feeding studies were performed between weeks 4 and 8. After week 8 on respective diets, rats were fasted overnight, and were injected the following morning (9–10 am) either leptin (4.94 nmol/kg, i.p.) or saline (400 µl, i.p.). After 1 h rats were deeply anesthetized with a mixture of sodium phenytoin and pentobarbital sodium (0.2 ml/100 g i.p., Beuthanasia-D Special C-III; Shering, Kenilworth, NJ) and tissue was collected.

Male Zucker rats (12 lean and 12 obese; 6 weeks old; Charles River) were fed chow for three weeks before tissue collection, during which time all feeding studies were performed.

### Feeding studies

Rats were fasted for 12 h starting at 9am. CCK feeding study: CCK (0.22 nmol/kg or 2.19 nmol/kg; i.p.) or saline (400 µl, i.p.) were administered and food intake was recorded every 20 min for 2 h. The low dose of CCK was chosen based on work by Savastano and Covasa [10] demonstrating that DIO rats were insensitive to this dose of CCK. We selected a 10 times higher dose to show that DIO rats had reduced sensitivity to CCK rather than an inability to respond. Leptin feeding study: Leptin (4.94 nmol/kg; i.p.) or saline (400 µl; i.p.) were administered. Leptin dose was selected based on previously published work in our laboratory [4], [23]. Animals were allowed to consume 10 kcal of chow for 40 min; food intake was then recorded every 20 minutes for 2 h as previously described [4]. All rats within each group (LF, DR and DIO) were paired according to body weight; one rat in each pair received either vehicle or treatment.

### Peptides and drugs

Leptin (rat) was obtained from Sigma (St. Louis, MO). Cholecystokinin, CCK (octapeptide, sulfated) was purchased from Bachem (Torrance, CA).

### Cell culture

Nodose ganglia were dissected under aseptic conditions, desheathed, and digested for 120 min at 37°C in 3 ml of Ca^2+^ and Mg^2+^ free HBSS containing 6 mg collagenase type Ia (Roche Diagnostics, Indianapolis, IM) as previously described [46]. Cells were plated onto four-well chamber slides, and maintained in HEPES-buffered DMEM with 10% fetal calf serum at 37°C in 5.5% CO_2_, and media changed every 24 h. Cells were maintained in culture for 72 h, at which time they were transferred to serum-free medium for 1 h before treatment with CCK (0.01–10 nM) either alone or in combination with leptin (10 ng/ml) for 2 h. Doses were selected based on previous publications [23].

### Immunohistochemistry

Cryostat sections of fixed nodose ganglia (5 µm) were mounted on polysine-coated slides (Polysine; MenzelGlaser, Braunschweig, Germany) and processed for immunohistochemistry with an antibody raised against Y2, MCH1R and CB1 (Santa Cruz Biotechnology, Santa Cruz, CA). Cultured neurons were fixed in 4% paraformaldehyde in PBS (30 min RT) and processed for immunocytochemistry with antibodies raised EGR1 (Cell Signaling Technology, Danvers, MA). Secondary antibodies were used as appropriate and included donkey anti-rabbit immunoglobulin conjugated with AlexaFluor 488 and donkey anti-goat immunoglobulin conjugated with AlexaFluor 546 (Molecular Probes, Eugene, OR). Specificity of immunostaining was determined by omitting the primary antibody and by pre-incubation with an excess of appropriate peptide where available. Cultured neurons were mounted in Vectashield with 4′,6-diamidino-2-phenylindole (DAPI; Vector Laboratories, Peterborough, UK) for nuclear localization. Images were collected using an Olympus spinning disc confocal microscope (BX61 System, Olympus, Melville, NY). The quantification of neurons expressing CB1, MCH1R, or Y2 was made by counting 200–300 immunoreactive cell profiles in four sections per ganglion, selecting sections separated by 90 µm that passed through the full length of the caudal and mid regions of the ganglia. Results are expressed as immunopositive cells as a proportion of all neurons in the relevant region.

### Western blotting

As previously described _ENREF_47 [47], briefly, 10 µg of proteins were used, unless otherwise stated in the legends, samples were loaded into precast 10% BisTris gel and the gel was run for 50 min at 200 V. The proteins were transferred 1 h. Primary antibodies were left to incubate overnight (STAT3 phosphorylated at tyrosine 705 (D3A7) Ab and EGR1 Ab, Cell Signaling Technology). GAPDH was used as a loading control (14C10) rabbit mAb, Cell Signaling Technology). The film was analyzed by Imagequant v 5.1 software (Amersham, Biosciences, Amersham, UK).

### Statistics

Statistical analysis was performed using Prism software (Prism 5.0, GraphPad Software Inc., La Jolla, CA, USA). Two way ANOVA was used to analyze energy intake data and leptin feeding studies (time and diet were used as the variables). One ANOVA was used to analyze immunohistochemical data. One way ANOVA was also performed to analyze western blot and CCK feeding studies with diet used as a variable and differences among groups were analyzed using multiple comparison procedure (Bonferroni). Differences were considered significant if p<0.05. Data are mean ± SEM. ^a,b,c^ different letters denote significant differences between groups. Unpaired one-tailed student's t-test was used to compare lean vs. obese Zucker rats' body weight and pSTAT3 expression in hypothalamus of these rats. Paired one-tailed student's t-test was used to compare the effects of CCK in LF, DR, and DIO rats. Significant differences were represented as * for p<0.05; ** for p<0.01; and *** for p<0.001.
